# Temperature-Biased miRNA Expression Patterns during European Sea Bass (*Dicentrarchus labrax*) Development

**DOI:** 10.3390/ijms231911164

**Published:** 2022-09-22

**Authors:** Maria Papadaki, Elisavet Kaitetzidou, Ioannis E. Papadakis, Dimitris G. Sfakianakis, Nikos Papandroulakis, Constantinos C. Mylonas, Elena Sarropoulou

**Affiliations:** 1 Institute of Marine Biology, Biotechnology, and Aquaculture, Hellenic Centre for Marine Research, 71003 Heraklion, Crete, Greece; 2 Department of Biology, University of Crete, 71409 Heraklion, Crete, Greece

**Keywords:** temperature, miRNA, European sea bass, development, gene expression, phenotypes, sex determination and differentiation, immune response

## Abstract

Environmental effects and, particularly, temperature changes have been demonstrated to influence the activity, function, and well-being of teleosts. Temperature may change seasonally in the wild, and in captivity under aquaculture operations. Moreover, climate change is expected to shift temperature profiles worldwide. MicroRNAs (miRNA) are important temperature-sensitive gene-expression regulators acting at the post-transcriptional level. They are known to be key regulators in development, reproduction, and immune responses. Therefore, early larval development of the European sea bass (*Dicentrarchus labrax*), one of the most extensively cultured species in Mediterranean aquaculture, was investigated at early rearing temperatures, i.e., 15, 17.5, and 20 °C, in regard to the impact of temperatures on miRNAs through sncRNA high-throughput sequencing but also at the phenotypic level in terms of growth, sex, vision, and skeletal deformities. Expression profiling revealed stage- and temperature-specific miRNA expression targeting genes with roles in reproduction and immune response mainly at the flexion and all-fins stages. Similar stage- and temperature-specific results were also observed concerning the number of rod cells and lower jaw elongation. The present work presents for the first time highly promising results on the influence of early rearing temperature at the post-transcriptional level during European sea bass development, with a putative impact on reproduction and immune response, as well as regarding teleost vision and larval development.

## 1. Introduction

In poikilothermic animals, temperature plays a vital role in the organisms’ physiology [1]. Temperature shifts beyond the accustomed temperature range may have undesirable impacts on the development [2,3], the reproduction [4,5], and the immune system of the animal [6,7,8]. Consequently, it is expected that the global warming effect, crucial especially in the Mediterranean region, which has been shown to have a faster warming rate than the global average and already a seawater temperature increase of 0.4 °C, may have an impact on the reproductive system, the development, and the well-being of the fish and thus an implication for the fish population dynamics. The importance of temperature alterations has been shown on the phenotypic level, e.g., in the European sea bass (*Dicentrarchus labrax*) [3], and in the same species also at the epigenetic level in regard to sex determination and reproduction [9]. Concerning the influence of temperature on early development at the epigenetic level, first approaches have been published regarding, e.g., the Atlantic cod (*Gadus morhua*) [2]. The influence of temperature during early development in relation to the immune response has been demonstrated in the model fish species zebrafish (*Danio rerio*) [7]. The interactions between the reproductive system and the immune system on the other hand have been reviewed by Lutton and Callard [10], but up until today, only scarce information has been generated here. The European sea bass, a broadly cultured species in the Mediterranean Sea, belongs to the well-studied teleosts, influenced by early-life rearing temperatures [3,9,11,12]. In this species, it has been shown that temperature during early larval development may have a strong influence on the development of skeletal deformities and the swimming capacity of the fish [3]. Thus, any failure during key developmental events may lead to malformations, developmental delays, and poor growth, but also to massive mortalities. In general, teleost embryonic and larval stages represent one of the most critical periods to guarantee high performance and superior quality in the successful developmental phases of the life cycle [13].

Besides the significance of monitoring early-life rearing during aquaculture practices to ensure the production of high-quality and healthy juveniles for further growth, controlling sex in various fish species is of great importance. This is primarily because in some species, one sex may grow better than the other, or reproduce at a later age, thus offering significant advantages for aquaculture rearing [3,14]. Concerning the European sea bass reared in aquaculture, it has been demonstrated that sex differentiation is based on a temperature-dependent sex determination system (TSD), and higher temperatures lead to an excess of males [5,15,16,17,18]. On the molecular level, regulatory control and response to temperature variations may occur during the process of transcription, post-transcription, translation, and post-translation [19]. Consequently, investigating epigenetics mechanisms may shed light on phenotypic changes triggered by temperature changes. Especially in terms of climate change, which results in increases in temperature, acidity, and salinity of the marine milieu, investigating the consequences at the epigenetic level may help to understand the regulatory mechanisms of adaptations to a new habitat due to environmental changes.

Epigenetics mechanisms comprise mostly DNA methylation and histone modifications but also small noncoding RNAs (sncRNA). While the former two act at the pretranscriptional level, the latter is known to regulate expression at the post-transcriptional level. Most of the studies so far have been focused on the changes in DNA methylation in relation to different environmental conditions and mainly in relation to temperature changes [20]. First indications supporting the involvement of sncRNA due to environmental changes have been presented in the Senegalese sole (*Solea senegalensis*) [21] and the Atlantic cod (*Gadus morhua*) [2], where alterations of several degrees in water temperature during early development resulted in different microRNA (miRNA) expression profiles. MicroRNAs belong to the most-studied class among small noncoding RNAs (sncRNA). They are small regulatory molecules with an average length of 22 nucleotides acting at the post-transcriptional level. It has been shown that miRNAs mainly bind at the 3′UTRs of their respective mRNAs, regulating gene expression largely through the repression of protein production by the inhibition of translation and mRNA degradation (reviewed in ref. [22]). Nevertheless, miRNA binding sites have also been found in other mRNA regions, including the 5′ UTR and coding sequence, and even within the promoter regions [23]. The binding of miRNAs to the 5′ UTR as well as to the coding regions may also have inhibiting effects on gene expression [24,25]. On the other hand, miRNA interaction with the promoter region has been reported to induce transcription [26]. Mature miRNAs, and specifically the seed regions, are normally highly conserved across species, but not all miRNAs are present in all species. In contrast, the conservation level of the target site within mRNAs among species is low. Mutations in miRNA binding sites or miRNA biogenesis regions have proven to weaken or even harm animal development [27]. miRNAs are known to be involved in a broad range of physiological processes, such as cell lineage specifications, reproduction, development, neurogenesis, stem cell differentiation, myogenesis, and immune response [28].

In the present work, we generated miRNA expression profiles to determine the involvement of miRNAs in four developmental stages (larval and post-larval) of European sea bass exposed to three different temperatures (15 °C, 17.5 °C, and 20 °C). The four developmental stages were further examined in terms of eye development as well as skeletal deformities after exposure to the three temperatures. Obtained miRNA expression patterns were compared to their respective phenotypes, and putative target genes were determined and grouped according to their pathways.

## 2. Results

### 2.1. Morphological Data

#### 2.1.1. Growth and Sex Ratios

At the end of the experiment, the mean total length was 284.23 ± 30.03 mm for the group reared initially at 15 °C, 324.33 ± 36.84 mm for the group reared initially at 17.5 °C, and 304.03 ± 34.72 mm for the group reared initially at 20 °C. The fish of the 17.5 °C and 20 °C groups had similar mean lengths, with the group at 15 °C exhibited similar values as the group at 17.5 °C, but lower values compared to the group at 20 °C.

The mean wet weight was 342.83 ± 96.75 g at 15 °C, 430.37 ± 118.69 g at 17.5 °C, and 435.17 ± 140.41 g for the group reared initially at 20 °C. Here, the group reared initially at 15 °C exhibited lower mean wet weight values than the other two groups, which exhibited similar values (ANOVA, Tukey’s HSD test, *p* < 0.05).

As far as the sex ratio of the three populations is concerned, only the group reared at 17.5 °C exhibited a sex ratio of about 1:1 (chi-squared test, *p* = 0.327), whereas the groups reared at 15 and the 20 °C exhibited sex ratios different than the expected 1:1 ratio (chi-squared test, *p* < 0.005), i.e., the male percentage was 64.1, 54, and 72.7% in the groups at 15, 17.5, and 20 °C, respectively (Table 1).

#### 2.1.2. Eye Development

**Figure 1:**
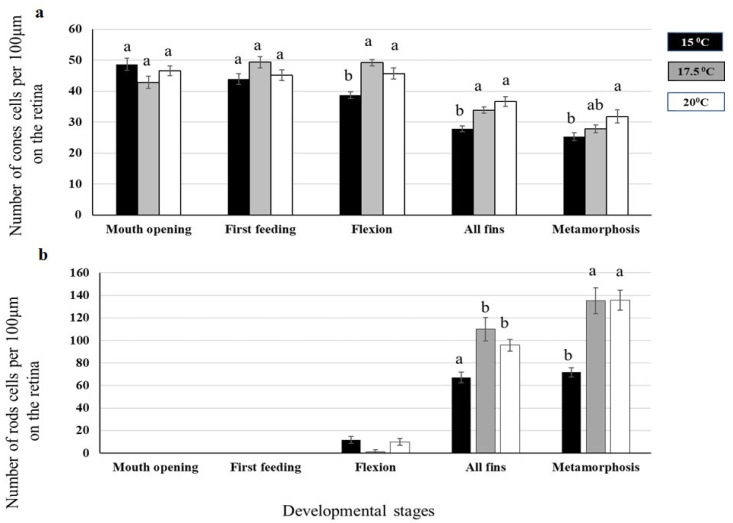
(**a**) Number of cone and (**b**) rod cells at five different developmental stages of European sea bass reared at three different temperatures (15 °C, 17.5 °C, and 20 °C) from the egg until the metamorphosis stage (n = 3). Different letters denote significant differences between experimental groups.

For the eye development analysis, the cone photoreceptors were already distinguishable at the mouth opening stage for all three rearing temperatures (Figure 1a), whereas the rods first appeared at the flexion stage at the rearing temperatures of 15 °C and 20 °C (Figure 1b, Supplementary Figure S1).

No significant differences in the mean number of cones were found between the different rearing temperatures at the stages of mouth opening and first feeding.

On the other hand, the mean number of cone cells was significantly increased (*p* < 0.05) at 17.5 °C and 20 °C at the developmental of flexion and all-fins stages. During the metamorphosis phase, a statistically significant difference (*p* < 0.05) in cone cells was only observed between the rearing temperatures of 15 °C and 20 °C. The rod cells were found to appear first at the flexion stage, in which the number of rod cells was measured to be higher at 15 °C and 20 °C, while a significant increase (*p* < 0.05) in rod cells number was detected for the all fins and metamorphosis stages at the rearing temperatures of 17.5 °C and 20 °C.

#### 2.1.3. Skeletal Deformities

**Figure 2:**
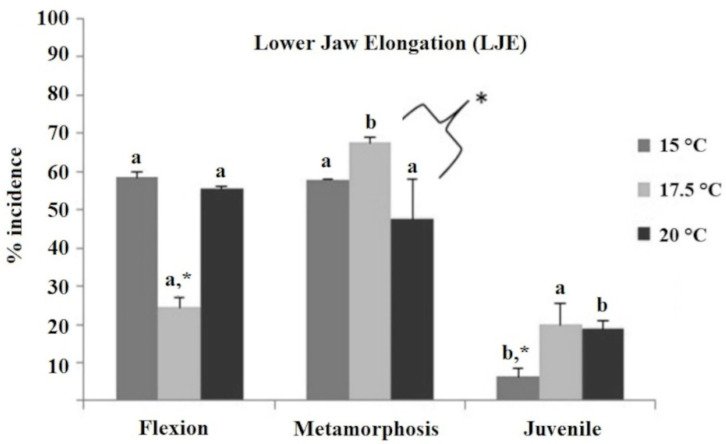
Incidence of lower jaw elongation (LJE) deformity (%) in European sea bass reared initially at three different temperature treatments (15 °C, 17.5 °C, and 20 °C) and evaluated at the flexion, metamorphosis, and juvenile stage. The existence of significant differences between the three temperature groups within each developmental stage (two-way ANOVA) is indicated by * (=*p* < 0.05). Different letters indicate significantly different means (two-way ANOVA, *p* < 0.05) between sampling times (flexion, metamorphosis, and juvenile) within temperature groups. Different letters denote significant differences between experimental groups.

The most common deformity encountered was the lower jaw elongation (LJE, Figure 2, Supplementary Figure S2), which was present at high frequencies in every temperature treatment and developmental stage, i.e., LJE was present at the flexion and metamorphosis stages in very high frequencies (48–68%), with the exception of the 17.5 °C treatment at the flexion stage, which was significantly lower than all the rest (25%, *p* < 0.05). On the other hand, the frequency of LJE at the juvenile stage was substantially lower compared to the other stages (ranging from 6 to 20%), while the 15 °C treatment was significantly lower than the other two temperature treatments (6 vs. 19 and 20%; *p* < 0.05).

### 2.2. sncRNA Data Generation and Corroboration

**Figure 3:**
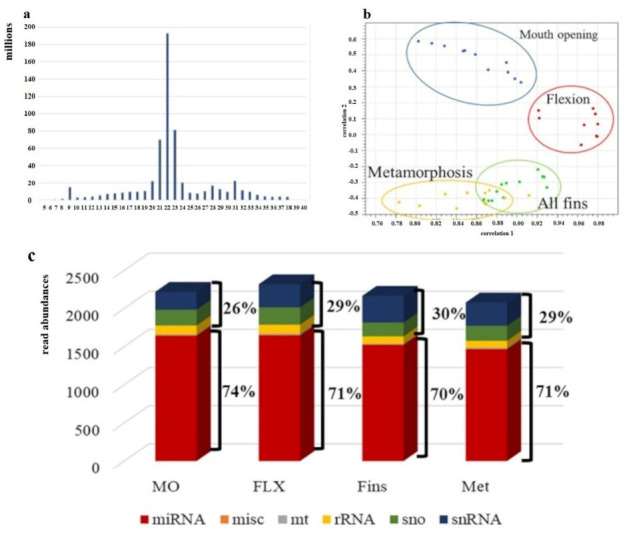
Evaluation of obtained sncRNA sequence reads. (**a**) Sequence length distribution. On the x-axes, the sequence lengths in nucleotides (nt) are plotted, and the y-axis shows the obtained number of counts of each sequence length. (**b**) PCA analysis of adapter- and quality-trimmed sequences according to their expression. (**c**) Annotation of sncRNA and their relative abundances are shown on the y-axis (n = 3–4).

Within this study, for each temperature, four developmental stages were sampled, resulting in four different sampling points. One time-point was still at the embryonic phase, while the other three were during larvae development. For each sampling point, four biological replicates were collected. About 215 million reads were obtained for each temperature, and an average of 13.6 million reads for each sample resulted in a total of ~654 million reads (Supplementary Table S1). Adaptor- and quality-trimmed sequences were plotted according to their read length, resulting in a length distribution with the main peak around 22 nt (Figure 3a). Expression profiling of the four developmental stages under study applying at least three replicates showed the expected stage-specific expression pattern (Figure 3b). Further classification of annotated reads, obtained out of all stages under study, showed that on average, 72% corresponded to miRNA and 28% to other sncRNA (Figure 3c).

### 2.3. Differential Expression

**Figure 4:**
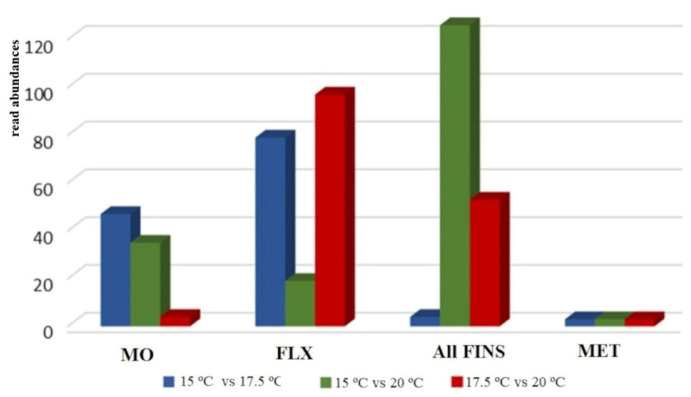
Overview of differentially expressed miRNA with significance threshold *p*-adj. < 0.05 and log2FC >|1| at the stages mouth opening (MO), flexion (FLX), all-fins (Fins), and metamorphosis (Met) (n = 3–4). The relative abundances are shown on the y-axis.

Differential expression analysis of reads annotated as miRNA and with a significance threshold of *p*-adj. < 0.05 and log2FC > |1| revealed a total of 86 miRNAs to be differentially expressed at the mouth opening stage, 195 miRNAs at the flexion stage, and 183 miRNAs and 9 miRNAs at the all-fins and metamorphosis stages, respectively. Comparing the expression profiles at the different temperatures, no common pattern can be observed across the four stages under study. Each stage appears to have a stage-specific pattern according to the different rearing temperatures. During mouth opening, a higher number of miRNAs were found for the temperature comparisons 15 °C to 17.5 °C and 15 °C to 20 °C, while for the flexion stage, the comparison of 15 °C to 20 °C seems to be the least-sensitive one. Regarding the all-fins stage, the highest sensitivity can be detected comparing temperatures 15 °C to 20 °C, while the metamorphosis stage seems not to be influenced significantly by different rearing temperatures (Figure 4).

#### 2.3.1. Differential Expression of Temperature at 15 °C and 17.5 °C

**Figure 5:**
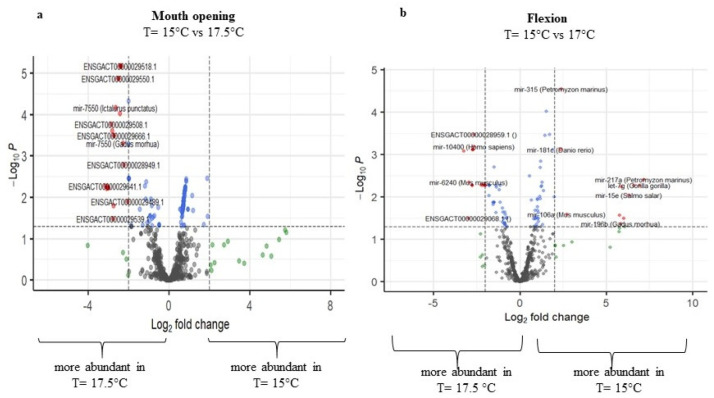
Volcano plots of transcript expression values at T = 15 °C and T = 17.5 °C at (**a**) mouth opening and (**b**) flexion stage. In grey color, not significant expression values are shown, in green expression, values with log2FC value > 2, in blue expression, values with *p*-adj. < 0.05, and in red expression, values with *p*-adj. < 0.05 and log2FC > 2 (n = 3–4).

Comparing the temperatures 15 °C and 17.5 °C, higher differences in miRNA expression were detected in two developmental stages, the mouth opening stage and the flexion stage, while the other two seemed not to be highly affected. Regarding the mouth opening stage, 157 miRNAs were regulated (*p*-adj. < 0.05), with 56 miRNAs being upregulated and 101 miRNAs being downregulated at 17.5 °C. Note that 121 miRNAs were annotated by the miRGamir database, and thus are unknown, while 36 miRNAs were annotated by miRbase. At the flexion stage, miRNAs with the aforementioned threshold were similarly up- (61) and down- (44) regulated at 17.5 °C (Figure 5a,b, respectively, and Supplementary Table S2), with miR-183 (*Tetraodon nigroviridis*), miR-196b (*Gadus morhua*), miR-125b-2 (*Salmo salar*), miR-27c (*Cyprinus carpio*), miR-15e (*Salmo salar*), miR-192 (*Tetraodon nigroviridis*), let-7g (*Gorilla gorilla*), and miR-217a (*Petromyzon marinus*) having zero read counts at 15 °C.

#### 2.3.2. Differential Expression of Temperature at 15 °C and 20 °C

**Figure 6:**
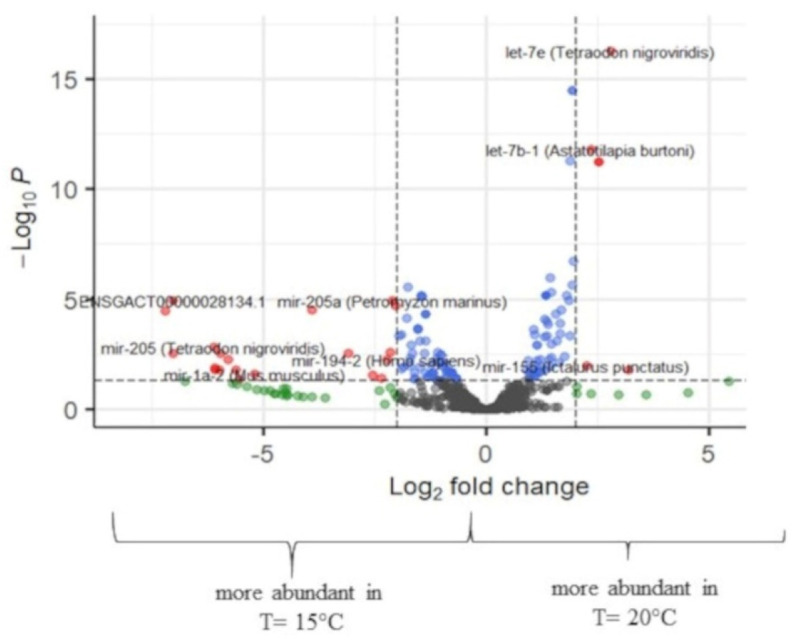
Volcano plot of transcript expression values at T = 15 °C and T = 20 °C at the all-fins. In grey color, not significant expression values are shown, in green expression, values with logFC value > 2, in blue expression, values with *p*-adj. < 0.05, and in red expression, values with *p*-adj. < 0.05 and logFC > 2 (n = 3–4).

The temperature difference of 5 degrees, i.e., 15 °C to 20 °C, showed the highest miRNA expression differences at the all-fins stage, with 137 miRNAs being differentially expressed (Figure 6), 79 miRNAs being upregulated, and 58 downregulated at 15 °C. Zero read counts at 15 °C were detected for 12 miRNAs. Mouth opening had a total of 38 differentially expressed miRNA, while 21 were differentially expressed at the flexion stage, and the metamorphosis stage did not have a significant number of regulated miRNAs (Supplementary Table S2).

#### 2.3.3. Differential Expression of Temperature at 17.5 °C and 20 °C

**Figure 7:**
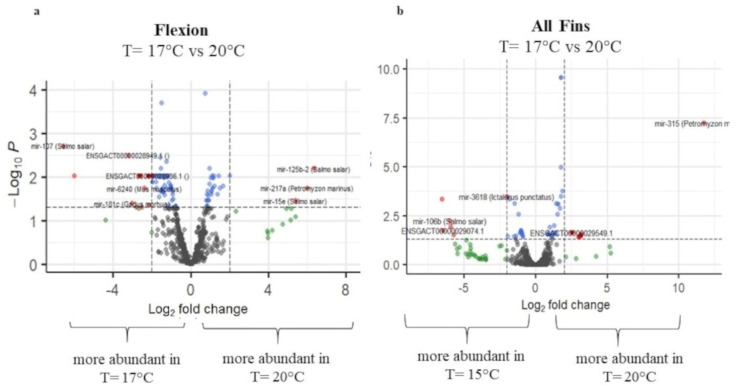
Volcano plots of transcript expression values at T = 17.5 °C and T = 20 °C at (**a**) flexion and (**b**) all-fins stage. In grey color, not significant expression values are shown, in green expression, values with logFC value > 2, in blue expression, values with *p*-adj. < 0.05, and in red expression, values with *p*-adj. < 0.05 and logFC > 2 (n = 3–4).

Concerning the comparisons between stages reared at the temperatures 17.5 °C and 20 °C, miRNA expression differences were detected for the flexion and all-fins stages. For the flexion stage, in total, 110 regulated miRNAs were detected, with 77 miRNAs found in higher abundance at 17.5 °C and 33 at 20 °C. Regarding the all-fins stage, 23 miRNAs were upregulated at 17.5 °C and 37 at 20 °C (Figure 7 and Supplementary Table S2).

### 2.4. Expression Analysis of Selected Genes Involved in Reproduction and Stress Response

**Figure 8:**
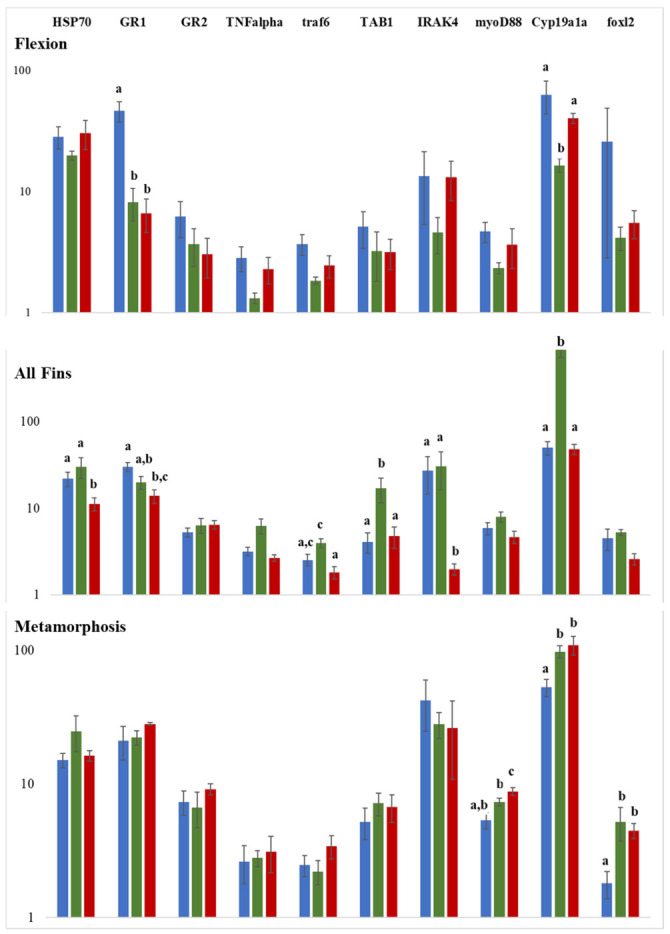
Gene expression analysis by qPCR of ten genes involved in reproduction and stress/immune response. Y-axes represent the relative gene expression on a log scale and x-axes the genes under study. Significance is indicated by different letter codes. Colors represent the mode of comparison, i.e.， blue 15 °C versus 17.5 °C, red 15 °C versus 20 °C, and green 17.5 °C versus 20 °C. The number of individuals tested was n = 4. Different letters denote significant differences between experimental groups.

To investigate the influence of temperature changes during reproduction and also to assess the putative stress/immune response at the molecular level, selected genes were studied by qPCR for three stages, flexion, all-fins, and metamorphosis. Following the miRNA expression study, the all-fins stage showed the highest expression response at a temperature shift of 15 °C to 20 °C (7 out of 10 genes studied), including the well-known stress indicator *hsp70* (Figure 8). Genes influenced by temperature at the flexion stage were glucocorticoid receptor 1 (*gr1*) and cytochrome oxidase 19a1a (*cyp19a1a*), while for the metamorphosis stage, three genes were detected to be temperature sensitive, i.e., *foxl 2* (forkhead transcription factor foxl2), *myoD88* (myeloid differentiation primary response 88), and *cyp19a1a* (*cyp19a1a* cytochrome P450, family 19, subfamily A). The expression of *cyp19a1a* was significantly downregulated at 17.5 °C compared to 15 °C and 20 °C, while at the metamorphosis stage, *cyp19a1a* was significantly upregulated at 20 °C.

### 2.5. Target Search and Panther Pathway Analysis

Lists of putative targets detected via RNAhybrid software of significantly differentially expressed miRNAs against the transcriptome downloaded from the Ensemble database are shown in Supplementary Table S3. Submitting target genes to the Panther Pathway analysis resulted in 13 statistically overrepresented pathways. Pathways involved in only one stage and one temperature shift are the Gonadotropin-releasing hormone receptor pathway, B cell activation, and oxidative stress response (all-fins 17.5 °C versus 20 °C), the wing signaling pathway (all-fins 15 °C versus 20 °C), and hypoxia response via HIF activation (flexion 15 °C versus 20 °C) (Table 2, Supplementary Table S4).

**Figure 9:**
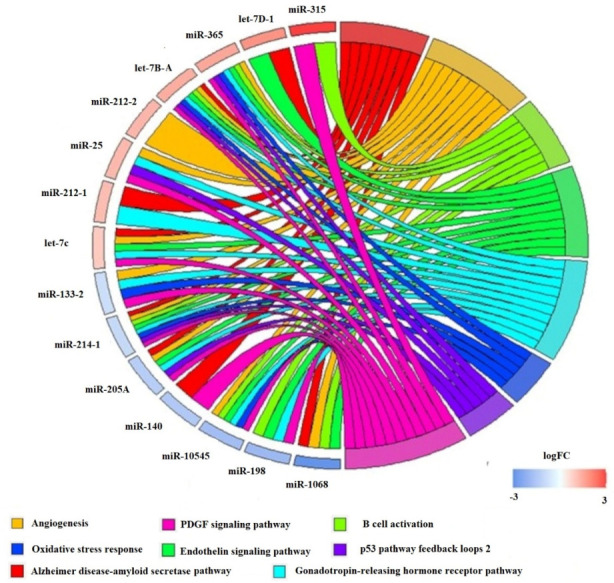
circPlot illustrating differential expressed miRNA at the all-fins stage comparing the treatments of 17.5 °C versus 20 °C linked to the Panther Pathways in blue miRNAs, which are downregulated at 20 °C, and in red, which are upregulated at 20 °C.

The pathway-corresponding miRNAs are provided in Supplementary Table S4, and for the comparison between all-fins 17.5 °C and 20 °C, for which most of the overrepresented pathways’ target genes were identified, a circPlot has been generated illustrating the involvement of particular miRNAs in the pathways. Particularly for the angiogenesis (P00005) pathway, 11 miRNAs were detected, for the B cell activation (P00010) 10, for the Endothelin signaling pathway (P00019) 4, for the Gonadotropin-releasing hormone receptor pathway (P06664) 10, the Oxidative stress response (P00046) comprised 8, the p53 pathway feedback loops 2 (P04398) 9, the Alzheimer disease-amyloid secretase pathway (P00003) 10, and the PDGF signaling pathway (P00047) 9 (Figure 9). No statistically significant pathways were found for the comparison of 17.5 °C versus 20 °C or 15 °C versus 20 °C at the mouth opening stage or the comparison of 15 °C versus 20 °C at the all-fins stage.

## 3. Discussion

The present work presents for the first time the impact of early rearing temperature at the post-transcriptional level during European sea bass development in association with morphological data. Based on the obtained results, responses to early rearing temperature as well as post-transcriptional regulation of immune response- and sex-related processes were stage-specific. It was demonstrated that earlier stages may not be as prone to temperature effects as the later developmental stages. The change in the number of structures of the retina rods and cones with respect to temperature may further indicate the different abilities of larval vision in relation to early life rearing conditions, which is not only an important finding for the aquaculture industry, but also has significant ecological implications. These visual element differences may be associated with a different perception of the prey items in the water column and the nutritional behavior of the larvae. Hence, from an ecological point of view, a possible change that may occur in sea temperature due to climate change may also alter the preying capacity and feeding habits of early-stage fish larvae.

Regarding the post-transcriptional gene regulation level by miRNAs, one of the utmost vital parameters to assure the best and most robust outcome and subsequent interpretation after sncRNA analysis is the obtained sequencing depth. The recommended sequencing depth of sncRNAs is between 5 and 10 million reads per sample, which has been achieved in the present study. It can further be assumed that at least 70% of the existing miRNAs were detected, according to the simulation experiments of Sun and colleagues [29], where they showed that around 70% of all expressed miRNAs may be found at a depth of 10 million reads when miRNAs with an expression of more than 15 reads are targeted. Furthermore, the sequence length distribution of trimmed reads may indicate the successful generation of sncRNA libraries but also their presence in the tissues under study. Since mature miRNAs are typically between 21 bp and 23 bp [28] long, length distribution should show a peak around these sizes. This criterion was also met within the present work. As already reported in the European sea bass [30], global miRNA expression pattern during development is stage-specific, which has also been shown in the present work, where expression patterns including all treatments and stages were plotted on a correlation scatter plot. Hence, the generated data are suitable to further investigate the effect of temperature differences at each of the developmental stages under study. Overall, the most peculiar profile regarding the abundancy of differentially expressed miRNAs was found at the flexion stage. At this stage, fewer miRNAs were found to be differentially expressed when the rearing temperature difference was 5 °C, i.e., T = 15 °C and T = 20 °C. Similarly, looking at the number of rod cells and percentages of lower jaw elongation carried out within this study, the temperature differences of 5 °C did not differ significantly. On the contrary, significant difference was found at a rearing temperature difference of 2.5 °C. It can, therefore, be hypothesized that at this stage small temperature differences, rather than larger ones, may have a stronger influence on the development, or that low and high rearing temperatures may induce similar regulation of development. Regarding the mouth opening stage and the all-fins stage, opposite patterns have been detected, indicating that when the larvae at the stage of mouth opening are kept at lower temperatures, it is not recommended to shift them to higher temperatures, while at the all-fins stage, it seems that the larvae are insensitive to temperature changes only between 15 °C and 17.5 °C. Nevertheless, one of the miRNAs being differentially expressed between 15 °C and 17.5 °C as well as between 15 °C and 20 °C at the all-fins stage is the well-studied miR-451, known as erythromiR, promoting the maturation of red blood cells [31,32,33]. The dependency of erythrocytes on thermal stress has been shown in the common carp (*Cyprinus carpio*) [34], where high temperatures were claimed to stress the fish by decreasing the red blood cells and hemoglobin. In the present work, miR-451 is downregulated at 15 °C compared to 17.5 °C and 20 °C; hence, the low temperature (15 °C) seemed to stress the fish at the all-fins stage and also at the flexion stage. Previous studies highlighted the function of fish erythrocytes (FE) in the phagocytic process. In contrast to human erythrocytes, FE are nucleated cells and contain several organelles and microtubule-associated proteins in the cytoplasm [35,36]. Mature FE have further been shown to play an important role in the fish immune response [34]. The importance of blood vessel generation and maturation (Angiogenesis) at different temperatures has also been reflected in the pathway analysis of putative targets being statistically overrepresented at the flexion and all-fins stage at different temperatures.

Among the strongest temperature-dependent regulated miRNAs in three of the stages under study, i.e., flexion, all-fins, and metamorphosis, was the miR-315. While at the flexion stage, miR-315 is upregulated at 15 °C, at the all-fins and metamorphosis stage, it is upregulated at 20 °C. Information published for this particular miRNA is, however, still very scarce. In shrimp, it has been hypothesized that higher levels of miR-315 facilitate the intrusion of the white spot syndrome virus [37], while in the fruit fly (*Drosophila melanogaster*), miR-315 affects the Wingless (Wg-Wnt) pathway, a highly conserved pathway among animals [38], which is known to regulate growth and tissue specification. The Wnt signaling pathway was also found to be statistically overrepresented at the all-fins stage when comparing the temperatures 15 °C to 20 °C, supporting the detected temperature dependency of the Wnt signaling pathway. Regarding the responsiveness to temperature in teleosts, more work has to be carried out to understand the role of miR-315 since the present work is the first to describe its temperature sensitivity.

At the level of gene expression in association with temperature, one of the well-known examples is the gonadal aromatase *cyp19a1a* gene. This enzyme converts testosterone to 17β-estradiol (E2), the main feminizing sex steroid in teleosts, and is expressed during early development before the histological differentiation of the gonads. *Cyp19a1a* was previously shown to have a temperature-dependent expression and hence to be involved in the temperature-dependent sex ratio shift in the European sea bass [9]. The latter authors have shown that a higher amount of *cyp19a1a* leads to higher female percentages. In the present study, the highest female percentages were found when fish were reared initially at 17.5 °C, and accordingly, significantly higher *cyp19a1a* expression at 17.5 °C was detected at the all-fins stage, while at 15 °C and 20 °C, *cyp19a1a* expressions was similar. On the other hand, at the flexion stage, the opposite pattern has been found. The all-fins stage also showed to be more sensitive to temperature changes in regard to the stress indicator gene *hsp70*, which was downregulated at 20 °C, compared to 15 °C and 17.5 °C. In addition to *hsp70*, other stress- and immune-related genes were differentially regulated, and thus, temperature changes mainly toward the lower temperature at this stage seem to stress the fish. It has also been reported that *gr1* (and not *gr2*) responds to stress in the European sea bass as well as in the gilthead sea bream (*Sparus aurata*) [39]. In the present study, lower temperatures at the flexion stage and at the all-fins stages showed higher *gr1* abundance compared to 20 °C, supporting the previous assumptions of putative stress at lower temperatures.

**Figure 10:**
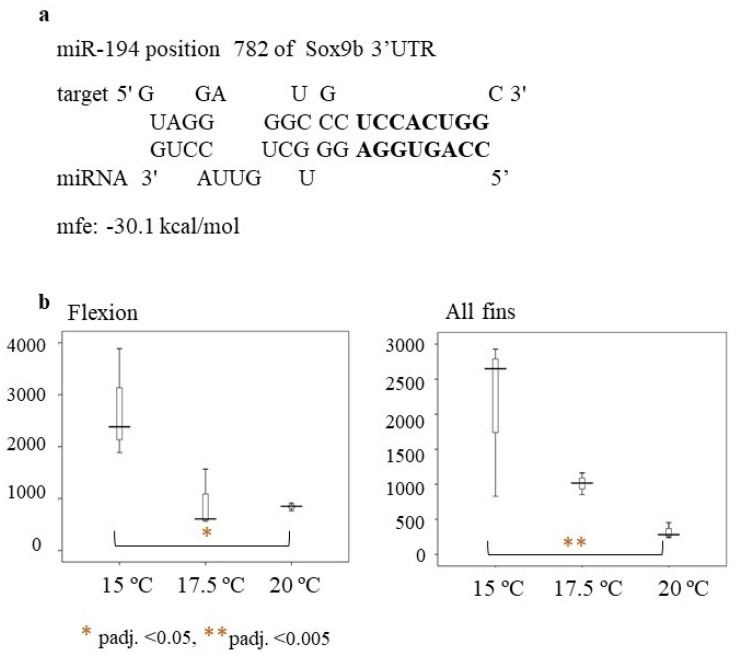
(**a**) Hybridization of miR-194 to Sox9b (ENSDLAT00005040776) of the European sea bass (**b**) Box-whisker plot of miR-194 relative expression at the flexion and all-fins stage. On the y-axes are the number of read counts. Significant differences are indicated by a red asterisk as follows: * = *p*-adj. < 0.05 ** = *p*-adj. < 0.005.

Recently, other genes, such as *sox3* and *sox9b*, have been shown to be linked to masculinization in the European sea bass and were repressed at lower temperatures (i.e., 16 °C) at the flexion stage [40]. MicroRNA analysis of the current work revealed that miR-194 may be a potential regulator of *sox9b*. Hybridization of the miR-194 seed region is perfect, with no mismatches from 2–9 bp, supporting the hypothesis of miR-194 regulating sox9b (Figure 10a). At the flexion stage and at the all-fins stages of the present study, miR-194 was significantly downregulated at rearing temperature T = 20 °C compared to rearing temperature T = 15 °C (Figure 10b). Thus, miR-194 may repress the *sox9b* expression at lower temperatures. The male percentages of the fish reared at 20 °C until full metamorphosis were also higher, supporting the hypothesis of miR-194 repressing sox9b at lower temperatures and indicating that at the flexion stage, and also at the all-fins stage, sex may be determined. This is in concordance with the finding of Geffroy and colleagues [12], who reported a significant upregulation of *sox9b* at 21 °C compared to 16 °C at the time of flexion. Taken together, these results may indicate the importance of the flexion and all-fins stages in relation to sex differentiation and temperature. However, this hypothesis has to be further investigated by more functional experiments, such as reporter gene assays.

In addition, at the all-fins stage, putative miRNA targets were found to be enriched in the Gonadotropin-releasing hormone (GnRH) receptor pathway. The role of GnRH in early development has been shown in several fish species, such as the gilthead seabream [41], the European sea bass [42,43], the zebrafish [44,45], and the cobia (*Rachycentron canadum*) [46]. Especially, in European sea bass, it has been shown that all different isoforms of GnRH appear long before gonadal differentiation, from 4–30 days after hatching [42], suggesting a role of GnRH in early developmental stages.

Additionally, after performing a knockout of the two GnRH genes in zebrafish, a function of GnRH on the brain, eye, and heart development was demonstrated [47]. Knockout of the gnrh3 gene in zebrafish leads to an increase in *sox9a*, *amh*, and *cyp11b* gene expression, favoring maleness [48]. This finding, together with the fact that miRNAs targeting the GnRH receptor pathway were differentially expressed at the all-fins stage in the present study, may lead to the assumption that GnRH could also be involved in the sex-differentiation process of European sea bass at an early developmental stage. Additional studies will be needed to investigate the specific role of GnRH in sex differentiation and the effect of temperature on its function.

## 4. Materials and Methods

### 4.1. Ethics Approval

Ethical approval for the study was obtained by the relevant Greek authorities (National Veterinary Services) under license No 255368. All procedures involving animals were conducted following the “Guidelines for the treatment of animals in behavioral research and teaching” [49], the ethical justification for the use and treatment of fishes in research: an update [50], and the “Directive 2010/63/EU of the European Parliament and the Council of 22 September 2010 on the protection of animals used for scientific purposes” (EU 2010).

### 4.2. Sampling

European sea bass eggs were collected on 14 February 2018, from a private fish farm in Sitia, Crete, Greece, and transferred to the aquaculture facilities of HCMR in Heraklion for larval rearing. Around 50,000 eggs were placed in each of six 500 L tanks (initial density of 100 individuals L^−^^1^), and developing larvae were reared until full metamorphosis at three different temperatures, 15, 17.5, and 20 °C, in duplicate tanks. Water temperature, pH, and dissolved O2 concentration were measured every day. Larvae were fed gradually with *Brachionus* spp., *Artemia* spp. (freshly hatched and enriched Instar II nauplii), and artificial feed (INVE Aquacultures S.A.) with some cofeeding during transitions as they grew in size. Phytoplankton Chorella sp. was added in the tanks during the first week of exogenous feeding. The photoperiod applied was 12 h light:12 h dark.

Samples of larvae were collected from both tanks and pooled at the following developmental stages: mouth opening (mouth opens, complete yolk sac absorption), flexion (beginning with the dorsal bending of the notochord tip concurrent with the development of the caudal-fin rays and supporting skeletal elements), all-fins (all-fins have been developed), and metamorphosis (end of the larval stage comprising acquisition of adult characters and loss of larval characters) (Supplementary Table S5) and preserved in RNA later at −80 °C until analysis. Samples at the same developmental stages were also collected for eye development evaluation and were preserved in 4% formaldehyde:1% glutaraldehyde [51] until histological analysis. For skeletal deformities evaluation during the samplings, which were conducted at the flexion and the metamorphosis stages, specimens were anesthetized with ethylene glycol-monophenylether (Merck, 0.2–0.5 mL l_ 1), fixed individually in phosphate-buffered 5% formalin [52], and stored in the dark at room temperature before staining. Staining for bone and cartilage followed a modification of the technique of [53]. In juveniles, X-rays of the populations were examined for deformities since staining techniques are mostly applied at the larval stages.

After reaching metamorphosis, fish were transferred to the nursery facilities, where they were kept in duplicate tanks at 19 °C until they reached around 1.5–2 g. Then, about 2000 fish were transferred to 2 m^3^ tanks. Length and weight measures (n = 15–20) were taken every two weeks until 193 dph, monthly until the first year of age, and every two–three months thereafter until the end of the experiment at 745 dph. Water temperature, pH, and dissolved O_2_ concentration were measured once per week. Fish density in the tanks was maintained at 4–5 kg m^−^^3^. Fish were fed with the use of self-feeders with pellets according to the size of the fish (Irida SA) until 1.5 years of age and manually thereafter.

Sexing of the fish was conducted through macroscopical examination of the gonads of 15–20 individuals at four different samplings (18 February 2019, 4 April 2019, 2 July 2019, and 6 March 2020 at 363, 408, 497, and 745 dph, respectively). The total number of fish sexed for each treatment group was 142 for the 15 °C group and 150 for both the 17.5 and 20 °C groups.

### 4.3. Rod and Cone Cells Analysis

Three larvae in each temperature condition were used for the histological analysis in every developmental stage. The samples were placed into the histological block horizontally in a dorsal position. For the quantitative determination of cones and rods in the retina, photographs were obtained from histological sections using a digital camera (Progres, Jenoptik AG, Jena, Germany) mounted on a Nikon Eclipse 50i microscope (Nikon, Melville, NY, USA) at a ×40 magnification. The analyzed photographs were taken from histological sections focused on each fish’s retina and the lens. The image-J analysis software (Rasband, W.S., ImageJ, U. S. National Institutes of Health, Bethesda, MD, USA, https://imagej.nih.gov/ij/, 1997–2018) was used to measure the lens’s diameter and count cones and rods manually.

### 4.4. Statistics

Differences in mean length and weight between the different temperature groups for the last sampling point were examined with one-way analysis of variance (ANOVA), having the rearing temperature as the changing parameter, followed by Tukey’s HSD test at *p* < 0.05. Statistical comparisons of the lower jaw elongation percentage and of the number of cones and rods in the eyes between groups were performed with the use of two-way ANOVA, having the rearing temperature and developmental stages as the changing parameters, followed by Duncan’s multiple range test, *p* < 0.05. Percentage data (% incidence of deformity) were arcsine square-root transformed before comparison. To test if the sex ratios (number of males:number of females) of the three populations were different from 1:1, a chi-squared test of independence was used, with α = 0.05 as a criterion for significance.

### 4.5. RNA Extraction and Evaluation

For total RNA extraction, 3 pools of larvae (at the mouth opening and flexion stages) and whole larvae (n = 3, at the stages of all-fins and metamorphosis) were disrupted in liquid nitrogen with mortar and pestle, and sample homogenization was achieved by passing the lysate five times through a 23-gauge (0.64 mm) needle. Isolation of total RNA comprising small RNAs was carried out with the use of the Nucleospin miRNA kit (Macherey-Nagel, Duren, Germany) following the manufacturer’s instructions. RNA quantity was estimated with NanoDrop ND-1000 spectrophotometer (NanoDrop Technologies, Wilmington, DE, USA), and the quality was further evaluated by agarose (1%) gel electrophoresis as well as by RNA Pico Bioanalysis chip (Agilent 2100 Bioanalyzer, Agilent Technologies, Santa Clara, CA, USA). Total RNA with RIN numbers > 8 were used for further downstream analysis.

### 4.6. sncRNA Libraries Construction and Sequencing

sncRNA libraries were generated from 1 μg of total RNA using the NEBnext multiplex Small RNA Library Preparation for Illumina sequencing (New England Biolabs, Ipswich, MA, USA). Size fraction was carried out as recommended by the manufacturer by running a polyacrylamide gel (6% TBE gel, Lonza, Basel, Switzerland) at 4 °C for 1 h. Each sample was tagged with a different multiplex identifier tag provided by NEB. Prior to sequencing, the generated sncRNA libraries were evaluated by DNA high-sensitivity chips (Bioanalyzer, Agilent). Quantification of the libraries was carried out with Qubit (Life Technologies, Carlsbad, CA, USA) in association with the results obtained by the DNA high-sensitivity chip run. A pool of the different indexed libraries at a concentration of 4 nM was single-strand sequenced over 4 lanes on the Illumina NextSeq sequencing platform at the Genomics Facility of the Institute of Molecular Biology and Biotechnology, Forth, Crete, Greece.

### 4.7. Sequencing Reads Analysis

Quality control of all reads was carried out applying the freely available Fastqc v0.10.0 software (http://www.bioinformatics.babraham.ac.uk/projects/fastqc). Sequencing reads were quality- and adapter-trimmed using Trimmomatic software 0.30 [54] and imported into the CLC genomics Workbench (v10.1). Putative sncRNAs were further extracted, and all reads were counted accordingly. The minimum sampling count (the number of copies of the raw sncRNAs reads included in the resulting count table) was set to 5. To annotate putative miRNAs, obtained sequencing reads were mapped against available teleost miRNAs in the miRbase (release 21.1) including Gorilla, Human and mouse in the following order: *Astatotilapia burtoni, Oryzias latipes, Tetraodon nigroviridis, Fugu rubripes, Danio rerio, Cyprinus carpio, Gadus morhua, Hippoglossus hippoglossus, Paralichthys olivaceus, Ictalurus punctatus, Salmo salar, Petromyzon marinus, Gorilla gorilla, Homo sapiens,* and *Mus musculus* [55] as well as against the three-spined stickleback (*Gasterosteus_aculeatus*) sncRNA database Gasterosteus_aculeatus.BROADS1.ncrna. CLC default parameters were used for allowed mismatches (2). Subsequently, the merging of variants of the same miRNAs was performed, generating a list of “sampled grouped” transcripts with the corresponding read count, which were normalized by transcripts per million (TPM).

### 4.8. Differential Expression Analysis

Differential expression (DE) analysis was carried out by submitting the obtained count matrix from the sample grouped merged annotated reads to SarTools version 1.2.0 [56] with default parameters. Transcripts with *p*-adj. < 0.05 and fold change (FC) >|1| were considered as differentially expressed. Heatmap and PCA analysis were carried out in R [57].

### 4.9. Quantitative Real-Time PCR (qPCR)

Quantification of mRNA expression was carried out by applying quantitative real-time PCR to profile the expression level of particular genes involved in reproduction and stress/immune response. Briefly, 1 μg of total RNA was reverse transcribed by using Prime ScriptTM RT reagent Kit with gDNA Eraser (Takara, Saint-Germain-en-Laye, France). qPCR reactions were carried out by applying Kapa SYBR fast qPCR Master Mix Kit (Kapa Biosystems, Sigma-Aldrich, Darmstadt, Germany). All real-time reactions were run on the Magnetic Induction Cycler (Mic) PCR Machine (Bio Molecular Systems, Upper Coomera, Australia). The specificity of amplification for each reaction was analyzed by dissociation curves using the micPCR Software supplied by Bio Molecular Systems. Ct values were obtained by submitting raw fluorescent data to the Miner algorithm [58]. mRNA expression was normalized to that of two reference genes, 18S ribosomal RNA (18s) and 40S ribosomal protein S30 (fau), using the 2^−ΔΔCT^ method. Levene test of variance was performed, followed by multiple comparisons applying the corresponding posthoc test, i.e., Tukey HSD or Tamhane. Additionally, a Kruskal–Wallis test was carried out to determine statistical significance.

### 4.10. Target Search

For target search, European sea bass 3′ UTRs were extracted applying the BioMart extraction tool from Ensembl. For the characterized differentially expressed miRNAs, putative targets were identified by applying RNAhybrid (version 2.12, [59]) with default parameters, while the energy threshold was set to mfe ≤ −30. Putative targets were reannotated by applying blasts against the GeneBank nr database of NCBI. For pathway analysis, Panther Pathways were chosen by applying the analysis type PANTHER Overrepresentation Test (Released 24 February 2021, PANTHER version 16.0 Released 1 December 2020). As a reference list, all genes in the database of the zebrafish (*Danio rerio*) were used, and a unique set of IDs were applied. The Fisher test was carried out using the FDR test as correction.

### 4.11. Data Access

Raw data have been submitted to the NCBI SRA database under the BioProject accession number PRJNA860319.

## 5. Conclusions

In summary, our results show that temperature influences the abundance of miRNAs during European sea bass larval and post-larval development. In particular, the flexion stage at 17.5 °C seemed to be less affected in comparison to 15 °C and 20 °C. Reduced miRNA dynamics have been seen at the stage of mouth opening, while none have been seen at the stage of metamorphosis, indicating that at the latter stage temperature may affect, to a lesser degree, proper embryonic, immune system, and sex development of the European sea bass. The regulation of *sox9b*, important for testis development, is suggested within the present study to be regulated by miR-194. Additionally, miR-194 has been found in a significantly higher copy number at 15 °C at the flexion and all-fins stage, which in turn may indicate the importance of both stages regarding the development of the sex of the fish. Furthermore, miRNAs identified to target genes involved in the GnRH receptor pathway were also found to be differentially expressed at the all-fins stage, and thus, GnRH could also be involved in the sex differentiation process of European sea bass at an early developmental stage. These highly promising results highlight the important roles of temperature-dependent miRNA regulation. Still, further investigations of specific candidates shown within the present work will be of importance to shed light on particular biological roles of miRNA acting at different temperatures during early rearing of European sea bass.

## Figures and Tables

**Table 1:** Percentages and ± standard deviation of European sea bass males and females at the adult stage, which were grown at three different rearing temperatures from the egg stage until metamorphosis. The difference to the expected 1:1 ratio was tested by the chi-squared test, *p* < 0.005.

	Male Percentage (%)	Female Percentage (%)
15 °C	64.1 ± 6.3%	35.9 ± 6.3%
17.5 °C	53.6 ± 4.2%	46.4 ± 4.2%
20 °C	72.7 ± 0.9%	27.3 ± 0.9%

**Table 2:** Panther Pathway analysis of putative miRNA target genes.

	Mouth Opening			Flexion			AllFins		
Statistical Overrepresentation of Pathways	15 °C vs. 17.5 °C	17.5 °C vs. 20 °C	15 °C vs. 20 °C	15 °C vs. 17.5 °C	17.5 °C vs. 20 °C	15 °C vs. 20 °C	15 °C vs. 17.5 °C	17.5 °C vs. 20 °C	15 °C vs. 20 °C
Alzheimer disease-amyloid secretase pathway (P00003)		No statistically significant results	No statistically significant results				No statistically significant results		
Angiogenesis (P00005)									
B cell activation (P00010)									
Cadherin signaling pathway (P00012)									
EGF receptor signaling pathway (P00018)									
Endothelin signaling pathway (P00019)									
FGF signaling pathway (P00021)									
**Gonadotropin-releasing hormone receptor pathway (P06664)**									
Oxidative stress response (P00046)									
p53 pathway feedback loops 2 (P04398)									
PDGF signaling pathway (P00047)									
Wnt signaling pathway (P00057)									
Hypoxia response via HIF activation									

## Data Availability

Raw data has been submitted to the NCBI SRA database under the BioProject accession number PRJNA860319.

## Supplementary Materials

The following supporting information can be downloaded at: https://www.mdpi.com/article/10.3390/ijms231911164/s1.
