# Supplementary material for: Temperature-Biased miRNA Expression Patterns during European Sea Bass (Dicentrarchus labrax) Development
Source: Int J Mol Sci. 2022 Sep 22;23(19):11164. doi: 10.3390/ijms231911164 (PMC9570215; doi:10.3390/ijms231911164)
Supplement: Supplementary file 1 [file ijms-23-11164-s001.zip › S5-Table.pdf]

International Journal of *Molecular Sciences*

MDPI

Supplemental table S5

Temperature-biased miRNA expression patterns during European sea bass (*Dicentrarchus labrax*) development.

Maria Papadaki[1,2], Elisavet Kaitetzidou[1], Ioannis Papadakis[1], Dimitris Sfakianakis[2], Papandroulakis N.[1], Constantinos C. Mylonas[1], Elena Sarropoulou[1*]

	Stage	Mouth opening	First feeding	Flexion	All fins	Metamorphosis
20 °C	Age (dph)	3	6	18	40	53
	Mean length (mm)	5,17 ± 0.14	5,53 ± 0.12	10,47 ± 1.19	21 ± 0.5	29,67 ± 3.43
17,5 °C	Age (dph)	5	9	20	47	62
	Mean length (mm)	5,53 ± 0.08	5,86 ± 0.15	10,94 ± 0.7	20,63 ± 0.79	27,35 ± 1.46
15°C	Age (dph)	6	11	30	53	86
	Mean length (mm)	5,41 ± 0.11	5,78 ± 0.13	12,09 ± 0.96	19,25 ± 0.76	29,10 ± 2.97
