# Supplementary figures and images for: Temperature-Biased miRNA Expression Patterns during European Sea Bass (Dicentrarchus labrax) Development
Source: Int J Mol Sci. 2022 Sep 22;23(19):11164. doi: 10.3390/ijms231911164 (PMC9570215; doi:10.3390/ijms231911164)

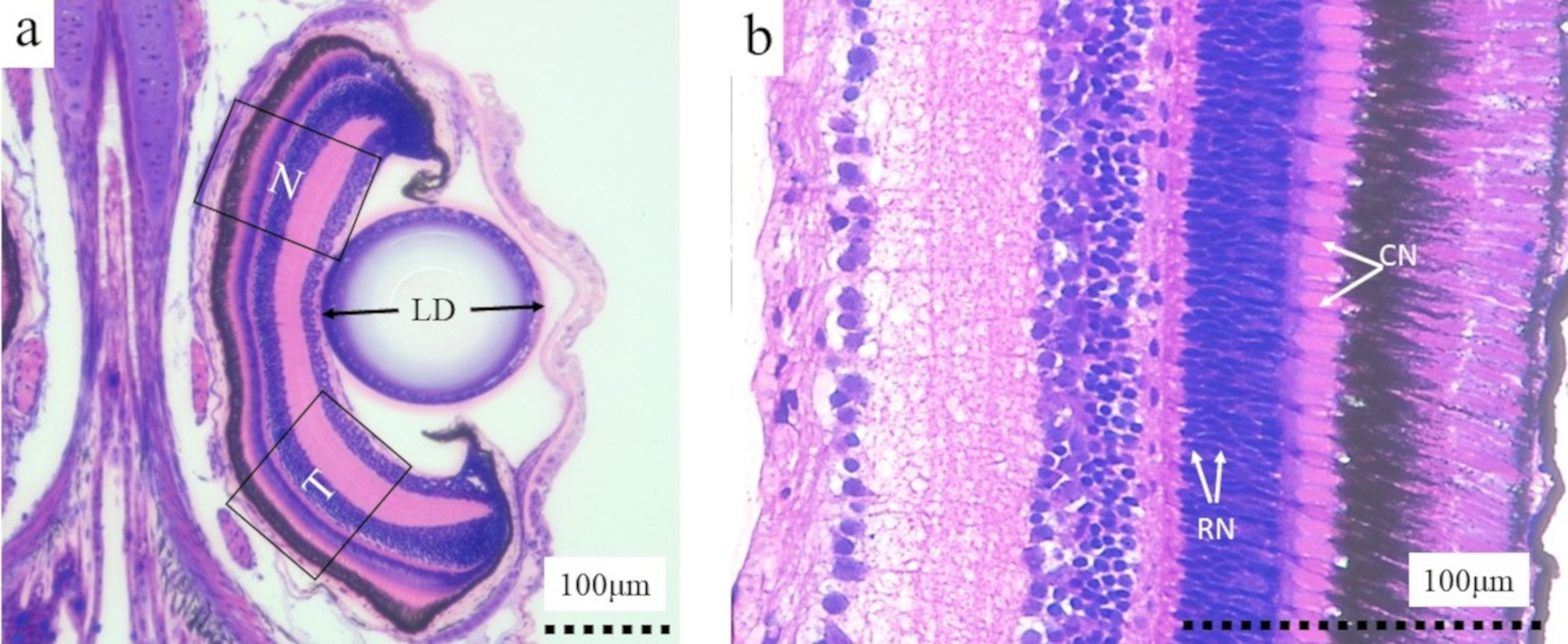

Supplement: Supplementary file 1 [file ijms-23-11164-s001.zip › S1-Figure.jpg]

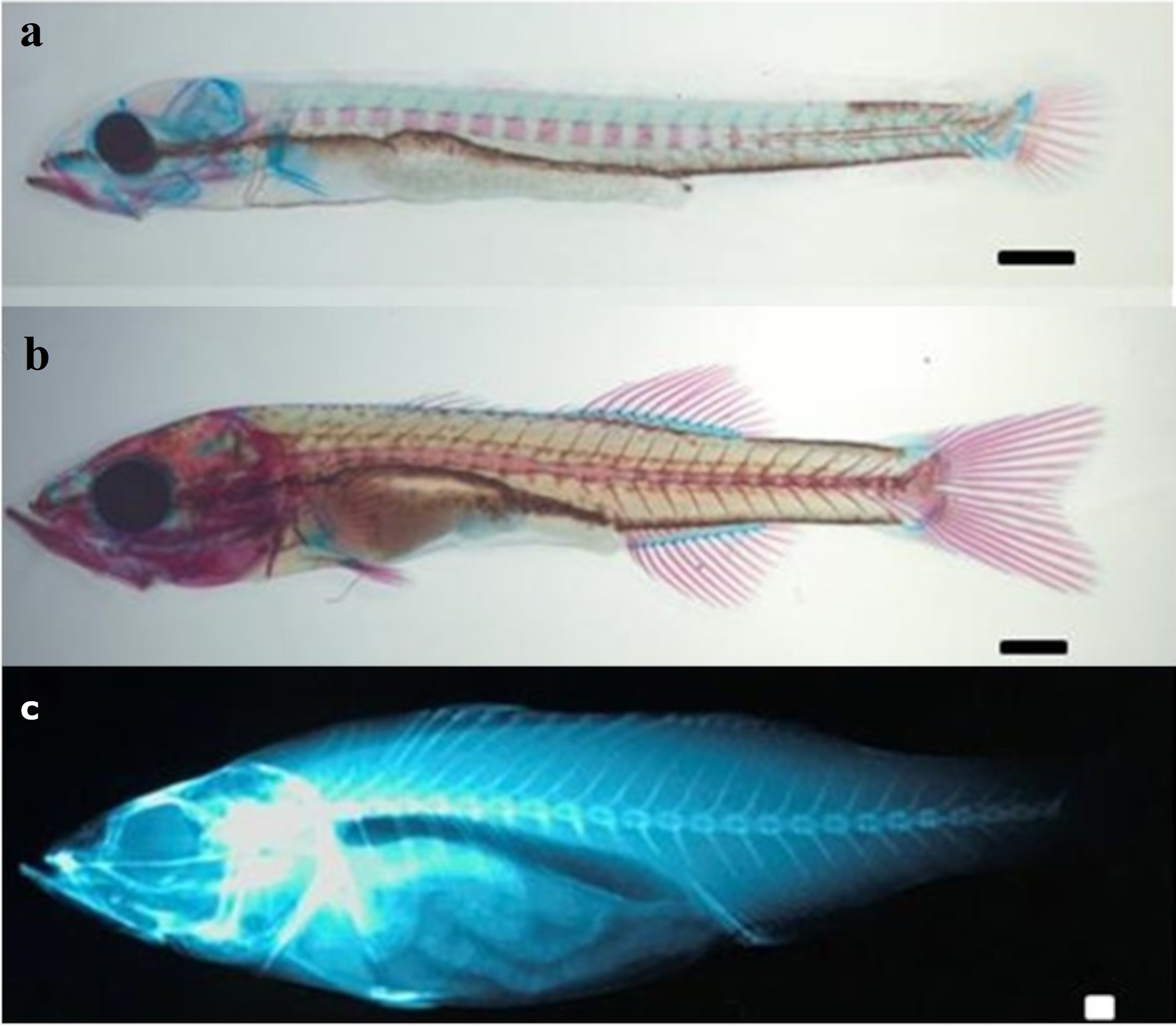

Supplement: Supplementary file 1 [file ijms-23-11164-s001.zip › S2-Figure.jpg]
